# TREM2 signaling pathway in sepsis-induced acute lung injury: physiology, pathology, and therapeutic applications

**DOI:** 10.3389/fmed.2025.1546292

**Published:** 2025-06-09

**Authors:** Hong-Lei Shen, Jing He, Fei Lin

**Affiliations:** Department of Anesthesiology, Guangxi Medical University Cancer Hospital, Nanning, Guangxi Zhuang Autonomous Region, China

**Keywords:** TREM2, sepsis, ALI, immunomodulation, therapeutic application

## Abstract

Sepsis emerges as a formidable and life-threatening condition, born from an unregulated immune response to infection, presenting a significant challenge to global health. A notable complication of sepsis is acute lung injury (ALI), marked by profound hypoxia, rampant inflammation, and the accumulation of fluid within the pulmonary system. ALI harbors the potential to escalate into acute respiratory distress syndrome (ARDS), thereby exacerbating the severity of sepsis. The triggering receptor expressed on myeloid cells 2 (TREM2), predominantly situated within various myeloid cell types, plays a pivotal role in the modulation of neurodegeneration, inflammation, neoplasms, and other pathologies. Recent investigations have illuminated TREM2's considerable involvement in septic lung injury; however, the precise mechanisms and therapeutic implications within this context demand further scrutiny. This article endeavors to elucidate the intricate interplay between sepsis, lung injury, and TREM2's role in immune modulation. It will furnish an overview of the TREM2 signaling pathway's functions and mechanisms in both physiological and septic lung injury scenarios, while also evaluating the current status and advancements in TREM2-targeted therapies.

## Introduction

Sepsis is characterized as a life-threatening condition triggered by infection that culminates in organ dysfunction (1). The clinical tableau presents a diverse array of symptoms, encompassing fever, tachycardia, tachypnea, and alterations in mental status. Absent prompt intervention, sepsis may rapidly escalate into septic shock, with the ominous potential for multiple organ failure (2). Epidemiological insights reveal a concerning rise in sepsis incidence each year, particularly within the confines of intensive care units, where it is estimated that a staggering 30% to 50% of patients may grapple with this affliction. Furthermore, as organ dysfunction deteriorates, the risk of mortality escalates significantly (3, 4). Thus, the imperative for early identification and management of sepsis becomes paramount, serving as a crucial determinant for enhancing patient prognosis and mitigating mortality rates.

Acute lung injury (ALI) frequently manifests in patients beset by sepsis, characterized by symptoms such as dyspnea, hypoxemia, and pulmonary infiltrates (5). The emergence of ALI within the sepsis context is intricately linked to the systemic inflammatory response. The release of inflammatory mediators precipitates damage to alveolar epithelial cells and engenders an increase in pulmonary capillary permeability, ultimately culminating in pulmonary edema and compromised gas exchange (6). The advent of ALI exacerbates the plight of sepsis patients, prolonging their hospital stays and inflating healthcare costs (7). Hence, a profound understanding of the mechanisms and etiologies underlying acute lung injury in sepsis is essential for the formulation of effective treatment strategies.

Triggering Receptor Expressed on Myeloid Cells 2 (TREM2) predominantly resides in microglia and peripheral macrophages. Since its identification in 2000, TREM2 has garnered recognition as a pivotal regulator of immune cell function (8). For instance, during the tumultuous course of sepsis and its associated acute lung injury, the activation of TREM2 amplifies phagocytic activity and cultivates an anti-inflammatory milieu, potentially mitigating the severity of injury (9). In athe realm of neurodegenerative diseases, TREM2 activation not only bolsters microglial survival and functionality but also enhances their phagocytic prowess (10). Moreover, an expanding body of evidence implicates TREM2 in the dynamics of tumor-associated macrophages (TAMs) and myeloid-derived suppressor cells (MDSCs), which conspire to forge an immunosuppressive tumor microenvironment, thereby influencing tumor progression and outcomes (11). Despite the considerable focus on the TREM2 signaling pathway's regulation of immune cells in neurodegenerative diseases and tumors, its role in peripheral inflammatory diseases remains largely uncharted.

This review endeavors to illuminate the role of TREM2 in the context of acute lung injury associated with sepsis. It aspires to elucidate the potential mechanisms at play. We systematically assess the expression levels of TREM2 in sepsis patients and scrutinize its correlation with clinical outcomes, drawing upon current research findings. Furthermore, we delve into strategies to harness TREM2 for the early diagnosis and intervention in septic lung injury, thereby offering novel insights for clinical treatment.

## TREM2 signaling pathway

### Molecular structure of TREM2

**Figure 1:**
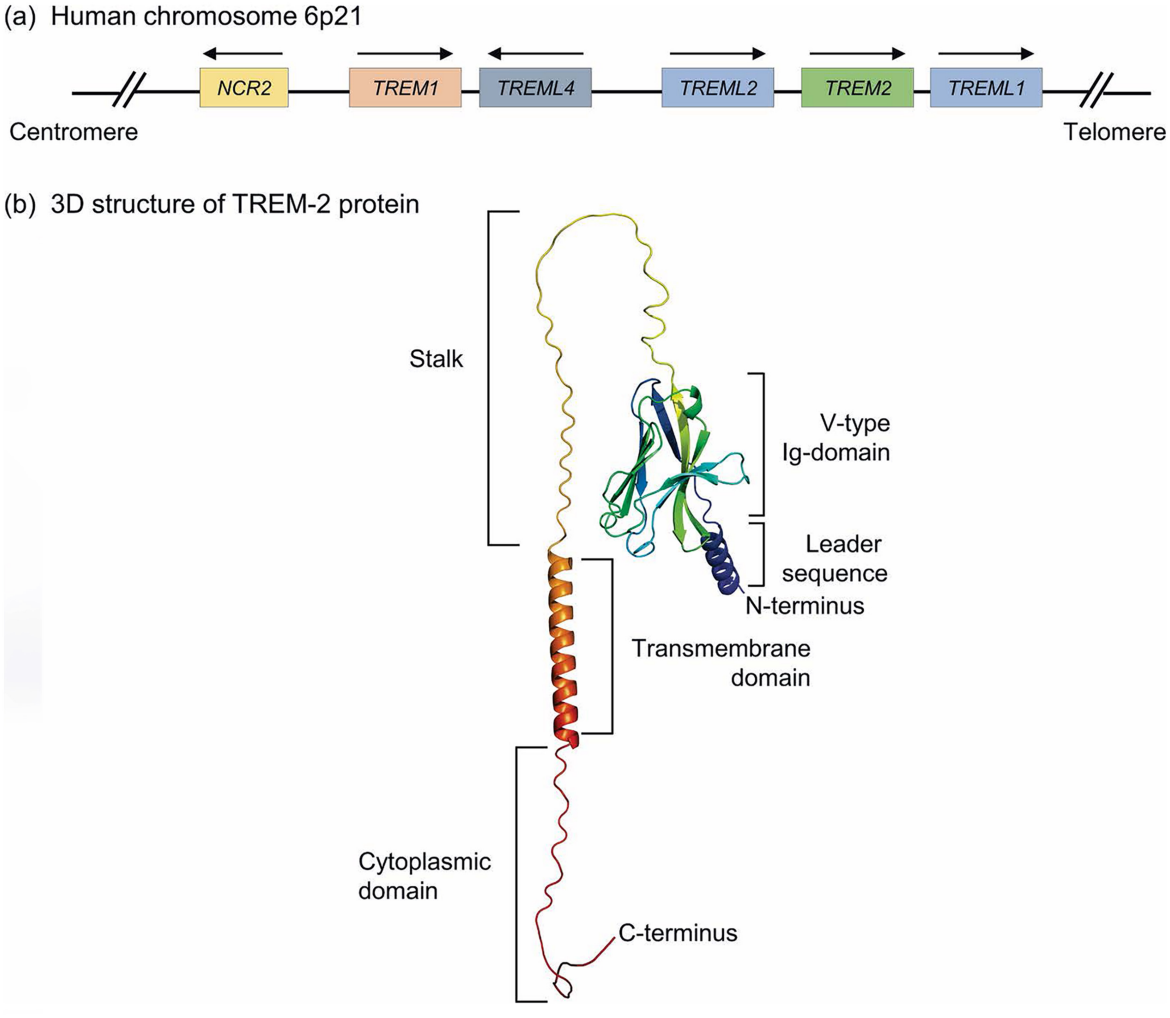
Genomic location and molecular structure of TREM-2 receptor. **(a)** Human TREM gene clusters located on human chromosome 6p21 locus, including TREM1, TREM2, TREML1, TREML2, and TREML4 genes. Also, this locus contains the NCR2 gene encoding NKp44, a typical cell-surface receptor of Natural killer (NK) cells and group 3 innate lymphoid cells. **(b)** A three-dimensional structure of the full-length TREM-2 protein, which is predicted by AlphaFold. Herein, the extracellular structure of TREM-2 contains a V-like Ig domain linked with a long stock followed by a TM domain. Also, the N-terminus leader sequence and C-terminus along with cytoplasmic domains are depicted in the predicted TREM-2 protein model (12).

TREM2, or triggering receptor expressed on myeloid cells 2, emerges as a transmembrane receptor belonging to the illustrious immunoglobulin superfamily, residing on human chromosome 6p21. The entirety of the gene spans 4,676 base pairs, intricately composed of five exons. These exons give rise to a glycoprotein predominantly localized within the microglia of the brain and peripheral macrophages. The architectural design of the TREM2 receptor encompasses a signal peptide, an extracellular domain replete with an immunoglobulin domain and a succinct handle sequence, alongside a transmembrane helix and a cytoplasmic tail. The extracellular domain plays a pivotal role in facilitating ligand binding, showcasing a highly glycosylated region that enhances its stability and functionality (12) (Figure 1). TREM2 engages with a diverse spectrum of ligands, encompassing various anionic molecules, whether free or affixed to the plasma membrane, including bacterial products, DNA, lipoproteins—such as low-density lipoprotein (LDL), apolipoproteins (APOE)—and phospholipids present in the body under normal physiological conditions (11). Investigations reveal that TREM2 undergoes proteolytic cleavage, yielding a soluble form (sTREM2) detectable in biological fluids, serving as an indicator of TREM2 activation and the cellular response to inflammation (13). The intricate structure of TREM2, coupled with the vast array of ligands, renders the prediction of its binding effects a complex endeavor. A profound understanding of the molecular architecture of TREM2 is imperative for the formulation of therapeutic strategies aimed at augmenting its function or compensating for its loss in pathological states.

### Activation of the TREM2 signaling pathway

Recent investigations have illuminated the intricate workings of TREM2, revealing its dual nature as both an independent entity and a participant in a multifaceted signaling network. This receptor is intricately woven into a tapestry of biological processes, encompassing phagocytosis, metabolism, cell survival, and anti-inflammatory responses, all of which culminate in transformative shifts in cellular phenotypes and functions. TREM2 emerges as a sentinel against the perils of unchecked inflammation, wielding the capacity to temper pro-inflammatory signaling cascades, particularly those orchestrated by toll-like receptors (TLRs). Through the downregulation of TLR4 expression, TREM2 plays a pivotal role in mitigating inflammatory responses, thereby safeguarding tissues from the ravages of acute inflammatory injuries (14). Emerging research suggests that TREM2 engages with adapter proteins DNAX-activating protein 10 (DAP10) and DAP12, utilizing oppositely charged residues within its transmembrane domain to forge heterodimers with either DAP10 or DAP12. The full-length TREM2 undergoes cleavage by ADAM17/10, yielding soluble TREM2 (sTREM2), which subsequently permeates the intercellular milieu to activate immune cells. Concurrently, γ-secretase cleaves the remaining carboxyl terminus of TREM2, facilitating the release of DAP12. This DAP12 then orchestrates the activation of tyrosine protein kinase Syk, setting in motion a cascade of tyrosine phosphorylation events that ultimately activate downstream mediators such as phospholipase Cγ2 (PLCγ2), phosphatidylinositol-3-kinase (PI3K), mammalian target of rapamycin (mTOR), and mitogen-activated protein kinase (MAPK), culminating in cellular activation. DAP10 complements this process by recruiting PI3K, thereby activating Akt and ERK to enhance signaling (15, 16). Recent findings have elucidated that TREM2-dependent phagocytosis necessitates the activation of the SYK/PI3K/AKT/PLCγ pathway, while the inhibition of NFκB activation by TREM2 operates independently of SYK, PI3K, and PLCγ activities. This study underscores the notion that TREM2 governs phagocytic activity through a pathway distinct from its anti-inflammatory function, thereby accentuating the receptor's multifaceted role within the realm of immune responses (17).

TREM2 orchestrates the migration of microglia toward sites of injury through the activation of specific signaling pathways, notably FAK (Focal Adhesion Kinase), Rac1, and Cdc42-GTPase. FAK, a pivotal molecule in cell adhesion and signal transduction, undergoes phosphorylation upon the activation of TREM2 receptors. This phosphorylation cascade subsequently activates Rac1 and Cdc42, leading to a profound remodeling of the cytoskeleton. Consequently, microglia are empowered to migrate with enhanced efficacy, responding swiftly to the exigencies of tissue damage and inflammation (18).

The signaling pathways engaged by TREM2, particularly those entwined with NF-κB, are of paramount importance in the orchestration of inflammatory responses, playing a critical role in both the initiation and resolution of sepsis (19). Following activation, TREM2 exerts an inhibitory influence on NF-κB activation by modulating intracellular signal transduction, thereby curtailing the expression of pro-inflammatory factors. This inhibitory mechanism serves to regulate the intracellular immune response, averting the perils of excessive inflammation and subsequent tissue damage. While TREM2 predominantly inhibits NF-κB activation, it is noteworthy that it can, on occasion, contribute to the emergence of inflammatory responses (17).

**Figure 2:**
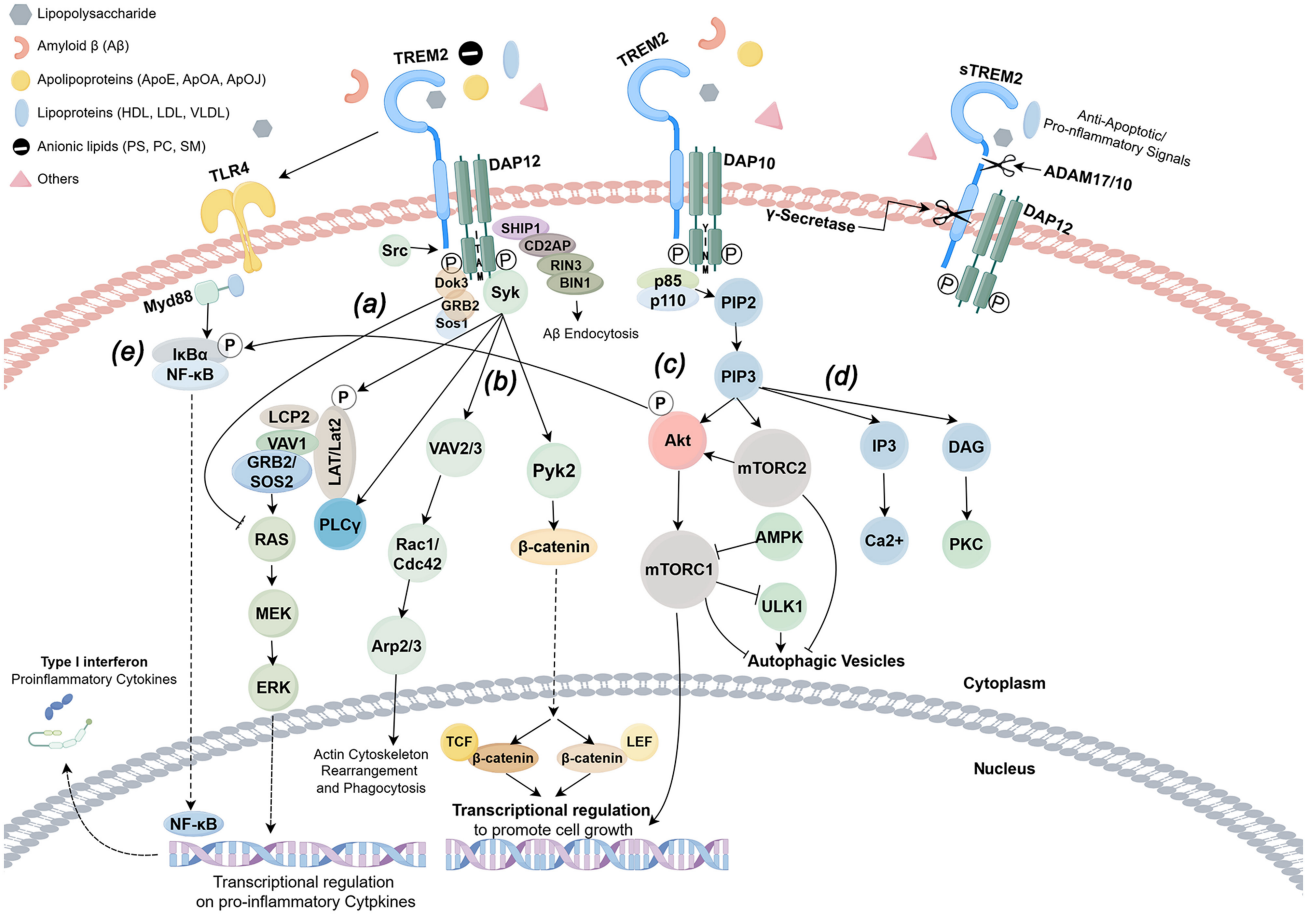
TREM2 signaling pathway (by Figdraw). TREM-2 orchestrates the activation of signaling cascades within myeloid cells through the engagement of DAP12, DAP10, and an array of other receptors upon the detection of ligands. Notably, DAP12 is endowed with an immunoreceptor tyrosine-based activation motif (ITAM), facilitating downstream signaling, whereas DAP10 is devoid of such a motif. The formation of heterodimers between TREM-2 and either DAP12 or DAP10 catalyzes the phosphorylation of adaptor proteins, thereby igniting a cascade of downstream pathways subsequent to ligand binding. **(a)** In the intricate signaling network of TREM-2/DAP12, the Src kinase activates DAP12, which in turn recruits the Sos1/2 and GRB2 junction proteins, leading to the inhibition of the RAS/MEK/ERK (or MAPK) pathway, a process that curtails the release of proinflammatory cytokines. **(b)** TREM-2 further establishes a complex with DAP12 or DAP10, engaging in crosslinking with receptors such as CSF1R, which modulates TREM-2/DAP12 signaling through the phosphorylation of DAP12/ITAM by Src kinase. This interaction recruits spleen tyrosine kinase (SYK), setting in motion downstream signaling pathways that involve guanine nucleotide exchange factors Vav2/3, Pyk2, and PI3K, with the assistance of DAP10. **(c)** Conversely, the interplay between DAP12 and DOK3 facilitates the recruitment of SHIP1, an inositol phosphatase characterized by a Src homology 2 (SH2) domain, which catalyzes the conversion of PIP3 to PIP2. This pivotal process governs the involvement and activation of intracellular signaling proteins associated with the membrane through PIP3. **(d)** The regulatory mechanisms of transcription are mediated by β-catenin and mTORC1. The activation of AKT and mTORC2 by PIP2/3 underpins metabolic homeostasis, while the RAS/MEK/ERK pathway modulates proinflammatory gene expression, with Ca2+ modulation facilitating actin remodeling. **(e)** TREM2 plays a crucial role in the dual responses of immune cells—both pro-inflammatory and anti-inflammatory—triggered by TLR4. In the nascent stages of bacterial infection, various antigenic substances can directly activate the TLR4 receptor, instigating the NF-κB signaling pathway that augments the expression of inflammatory factors. Concurrently, the TREM2-DAP12-SYK pathway activates the IKK complex, promoting the degradation of IκBα and enabling NF-κB to translocate into the nucleus, thereby driving the inflammatory response. Upon activation, TREM2 recruits SHP-1, a protein that dephosphorylates critical molecules within the NF-κB pathway, such as IKKβ and p65, thereby obstructing their nuclear translocation. This mechanism effectively inhibits the excessive activation of NF-κB instigated by TLR4, leading to a reduction in the release of pro-inflammatory factors such as TNF-α and IL-6. Furthermore, it curtails glycolysis in macrophages via the AKT/mTOR pathway, diminishing the production of pro-inflammatory factors reliant on NF-κB.

Moreover, TREM2 engages with a multitude of other signaling pathways, thereby influencing their functional dynamics. For instance, TREM2 is implicated in the deposition of Alzheimer's disease-associated Aβ plaques and tau proteins, thereby modulating the neurodegenerative process through the clearance of these pathological entities (20). Additionally, TREM2 plays a significant role in regulating the release of inflammatory mediators within macrophages, thereby shaping the inflammatory milieu of the local microenvironment (21). Furthermore, the activation of TREM2 is intricately linked to the NLRP3 signaling pathway, which modulates the inflammatory response of microglia in the context of elevated glucose levels. This connection suggests that TREM2 may serve as a crucial nexus between metabolic disorders and neuroinflammation (9) (Figure 2).

## TREM2 and immune cells

TREM2 finds its primary expression within a diverse array of myeloid cells, encompassing granulocytes, bone marrow-derived macrophages (BMDMs), monocyte-derived macrophages (MDMs), alveolar macrophages, and osteoclasts (OCs). Notably, TREM2 is also richly present in microglia, while its expression remains absent in lymphocytes (12). This differential expression of TREM2 across immune cell types underscores its pivotal role in orchestrating the immune response to a multitude of conditions, including various infectious diseases, Alzheimer's disease, and tumors (22–25). As contemporary research predominantly centers on peripheral macrophages and microglia, this section shall delve into the intricate role of TREM2 within these two cell types.

### TREM2 and macrophages

TREM2 emerges as a pivotal immune regulatory factor, intricately influencing the activation, polarization, phagocytosis, and metabolic processes of macrophages. Upon binding to its ligand, TREM2 initiates a cascade of intracellular signaling pathways, notably the PI3K/AKT pathway, which serves to bolster macrophage survival and proliferation (26). Empirical investigations illuminate TREM2′s role in attenuating the activation of the NF-κB pathway, a fundamental mechanism governing the synthesis of pro-inflammatory cytokines during inflammatory responses. This attenuation culminates in a reduction of inflammatory mediators such as TNF-α and IL-6, thereby mitigating the excessive inflammation that can precipitate tissue damage and chronic diseases (21). Furthermore, TREM2 curtails the inflammatory response by inhibiting the activation of the NLRP3 inflammasome, thereby diminishing the release of inflammatory factors (27). A further study elucidated that TREM2/β-catenin orchestrates macrophage pyroptosis by suppressing NLRP3 inflammasome expression, thereby enhancing the macrophage-mediated clearance of pyogenic bacteria (28).

Conversely, TREM2 possesses the capacity to steer the polarization of macrophages from a pro-inflammatory M1 phenotype toward an anti-inflammatory M2 phenotype, activating a myriad of downstream signaling pathways, including the NF-κB/CXCL3 axis, thus amplifying their anti-inflammatory effects and reparative capabilities (21, 29). Moreover, in models of kidney disease, the absence of TREM2 precipitates macrophage apoptosis and polarization via the JAK-STAT pathway, thereby exacerbating kidney injury (30).

In the realm of bacterial infection, TREM2 emerges as a pivotal enhancer of macrophage efficacy, orchestrating the clearance of pathogens and cellular debris through the regulation of their metabolic state. This modulation fortifies the antibacterial defenses of macrophages (19, 26, 31). The absence of TREM2 is intricately linked to a diminished capacity for bacterial clearance and an escalated vulnerability to infection, thereby underscoring its protective essence within macrophage functionality (26). Moreover, the deficiency of TREM2 incites a reprogramming of macrophages, which exacerbates inflammatory responses and hinders tissue repair, further highlighting its critical role in sustaining immune equilibrium (32). Compelling research has illuminated the role of the transcription factor P53 in the upregulation of TREM2 expression, thereby enhancing macrophage lipid metabolism, activity, and phagocytic prowess (33). In a murine model of sepsis intertwined with non-alcoholic fatty liver disease (NAFLD), TREM2 is instrumental in preserving the metabolic harmony between macrophages and hepatocytes, ultimately improving liver energy supply and outcomes in sepsis (34). Furthermore, TREM2 is intricately associated with steroidogenesis in adrenocortical cells during the tumultuous phase of lipopolysaccharide-induced septic shock (35).

In addition, TREM2 plays a vital role in tissue repair and regeneration by deftly modulating the metabolic status of macrophages. For instance, TREM2 facilitates the healing process following cardiac injury by influencing lipid metabolism within macrophages (36). The TREM2hi Mac1 cells actively engage in the clearance of dysfunctional mitochondria expelled from cardiomyocytes, thereby preserving the delicate homeostasis of cardiomyocytes and safeguarding the heart amidst septic conditions (37). Conversely, the absence of TREM2 can severely compromise macrophage functionality, culminating in exacerbated tissue damage and inflammatory responses, as demonstrated across various disease models (30).

In summation, TREM2 stands as a crucial regulatory molecule, intricately interacting with macrophages and shaping the immune response. Its influence on macrophage functionality is profound, playing a significant role in diverse pathological conditions and thereby affecting the overarching immune response.

### TREM2 and other immune cells

Regarding TREM2 and other immune cells, investigations indicate that the maturation of dendritic cells (DCs) mediated by TREM2/DAP12 can enhance partial T cell activation even in the absence of external antigens, a process that is critical for sustaining T cell homeostasis and survival (38). Additionally, the expression of TREM2 within nephritis DCs catalyzes the production of nitric oxide (NO), which subsequently inhibits the differentiation of Th17 cells and mitigates the progression of chronic kidney disease (CKD) (39). Moreover, it has been documented that TREM2 is abundantly expressed in CD4+ T cells amidst infection and inflammation. The TREM2 receptor complex incites a pro-inflammatory Th1 response through the activation of the STAT1/STAT4 pathway, thereby orchestrating the regulatory mechanisms inherent to adaptive immunity and host inflammatory responses (40). A multitude of studies have elucidated the extensive regulatory influence of mesenchymal stem cells on immune responses, revealing that the overexpression of TREM-2 significantly curtails the production of inflammatory cytokines, such as TNF-α and IL-1β, while simultaneously fostering the elevated secretion of the anti-inflammatory cytokine IL-10 (41). Nevertheless, the intricate mechanisms underlying these processes remain to be thoroughly explored. In a separate investigation concerning tumor immunity, it was discovered that ApoE, released by prostate tumor cells, engages with the immunosuppressive TREM2+ neutrophil subpopulation—also recognized as polymorphic nuclear myeloid-derived suppressor cells (PMN-MDSCs)—thereby promoting its senescence (42). This finding prompt contemplation regarding the potential existence of analogous regulatory mechanisms in inflammation-related diseases, which may significantly influence immune responses.

The aforementioned summary fully underscores the vital biological functions of TREM2 across a spectrum of immune cells, particularly within macrophages, microglia, and dendritic cells. Its regulatory scope encompasses phagocytosis, polarization transitions, cell survival, and proliferation, as well as antigen presentation. In pathological states such as sepsis, the role of TREM2 is indispensable for immunomodulation and tissue repair. Thus, delving into the mechanisms of TREM2 across diverse immune cells is paramount for unraveling the complexities of sepsis-induced lung injury and for identifying novel therapeutic targets in clinical practice.

## TREM2 and sepsis-induced acute lung injury

### About sepsis-induced acute lung injury

Sepsis is a systemic inflammatory response syndrome caused by infection, and its pathophysiological changes involve complex responses of multiple systems. The genesis of sepsis unfolds with the invasion of pathogens, accompanied by the aberrant activation of the host immune response, culminating in the tempest of a cytokine storm. Upon the onset of infection, the host immune system, through the vigilant eyes of pattern recognition receptors (PRRs), including the toll-like receptors (TLRs), discerns the presence of pathogens. This recognition of pathogen-associated molecular patterns (PAMPs) ignites the activation of macrophages and other immune sentinels, setting in motion an inflammatory cascade that releases a plethora of inflammatory mediators, such as tumor necrosis factor (TNF) and interleukins (IL-6, IL-1β), among others. These inflammatory agents exert a profound influence on local tissues, instigating endothelial dysfunction and amplifying vascular permeability. Moreover, they can incite systemic inflammatory reactions coursing through the bloodstream, leading to organ dysfunction, with acute lung injury standing as a particularly grievous consequence (19, 43, 44). Furthermore, recent investigations illuminate the pivotal roles of mitochondrial dysfunction and oxidative stress, suggesting that compromised mitochondrial function may precipitate energy deficits within the cells of those afflicted by sepsis (34, 45).

Sepsis-induced Acute lung injury (SI-ALI) is a prevalent and harrowing complication of sepsis, manifesting as a severe respiratory affliction characterized by profound hypoxemia, an unchecked inflammatory response, and the accumulation of both alveolar and interstitial edema, potentially culminating in the development of acute respiratory distress syndrome (ARDS). The pathophysiological underpinnings of SI-ALI are multifaceted, with the release of inflammatory mediators serving as a critical catalyst in the onset of acute lung injury (46). This cascade of events can inflict damage upon the alveolar-capillary membrane, heightening permeability and leading to pulmonary edema, thereby impairing gas exchange (47). In addition, the activation of inflammatory mediators incites a response from other immune cells residing within the alveoli and endothelial cells, compelling them to secrete an array of chemokines and cytokines, thus perpetuating a vicious cycle that exacerbates lung injury (48). For example, the infiltration of neutrophils into the pulmonary landscape serves as a hallmark of sepsis-induced lung injury, wherein activated neutrophils unleash reactive oxygen species (ROS) and proteolytic enzymes, further intensifying tissue damage (49, 50).

Moreover, pyroptosis—a distinct form of programmed cell death—plays a crucial role in the context of septic lung injury. Recent studies have elucidated that the activation of the NLRP3 inflammasome within lung epithelial cells triggers the release of inflammatory cytokines, culminating in further detriment to lung tissue (51, 52). Thus, the role of inflammatory mediators is indispensable in the progression of septic lung injury. A comprehensive understanding of the regulatory mechanisms governing these mediators may pave the way for mitigating the inflammatory response and curtailing organ damage, ultimately enhancing the prognosis for patients grappling with septic lung injury.

### Mechanism of TREM2 in SI-ALI

TREM2 assumes a dual role within the intricate tapestry of the inflammatory response, nurturing anti-inflammatory processes while simultaneously providing a protective shield amidst the tumult of inflammation. As a central figure in immune regulation, TREM2 has captured the attention of researchers delving into the complexities of septic lung injury. Compelling evidence indicates that TREM2 tempers excessive inflammatory responses through the nuanced modulation of macrophage activity and functionality, thereby alleviating the burdens of sepsis-induced acute lung injury. For instance, the activation of TREM2 can catalyze a transformation in macrophages toward an anti-inflammatory phenotype, culminating in a reduction of pro-inflammatory factor release and a diminishment of the pulmonary inflammatory response (26). Moreover, TREM2 fortifies the immune response against sepsis by engaging its ligand and activating signaling pathways that bolster macrophage survival and functionality (53).

Recent studies illuminate that Rhein fosters M2 polarization in macrophages by targeting the NFATc1/TREM2 pathway, a mechanism pivotal in regulating the inflammatory response and prognosis following acute lung injury (54). Galectin-3 emerges as a significant upstream factor implicated in lung ischemia-reperfusion injury (LIRI), and within this framework, the enhancement of TREM2 expression reveals potential in mitigating lung injury (55). In addition, grape seed proanthocyanidins orchestrate M2a macrophage polarization through the TREM2/PI3K/Akt pathway, thereby ameliorating LPS-induced acute lung injury (56). Related investigations underscore that plasma sTREM2 may serve as a prognostic marker in the assessment of acute lung injury, intricately regulating inflammation alongside the downstream TREM2 response (57). Furthermore, the vasoactive intestinal peptide has been demonstrated to recalibrate the TREM-1/TREM-2 ratio within lung cells, influencing the onset of acute lung injury (58).

TREM2, conversely, orchestrates the antimicrobial defense mechanisms of macrophages, fortifying their survival and enhancing their functionality amidst the presence of pathogens. For instance, the absence of TREM2 is linked to compromised bacterial clearance and an escalated vulnerability to infection, thereby underscoring its pivotal role in the protective effects against sepsis (26). Moreover, TREM2 influences the metabolic interplay between macrophages and hepatocytes, a critical factor in sustaining homeostasis during the tumultuous state of sepsis (34). Intriguingly, a study concerning acne has posited that squalene elevates the expression of TREM2 within macrophages residing in lesions, thereby augmenting their phagocytic capacity to engulf lipids and bacteria. Nevertheless, given that squalene possesses the ability to scavenge reactive oxygen species (ROS) and downregulate the enzymes implicated in ROS generation and its subsequent reactions, it inadvertently curtails the antibacterial efficacy of TREM2-expressing macrophages, resulting in a notable deficiency in antibacterial functionality (59).

Further investigations have illuminated that in the context of sepsis-induced acute lung injury in murine models, TRIM21 (tripartite motif-containing protein 21) attenuates the expression of IRF1 (interferon regulatory factor 1) through the mechanism of ubiquitination, thereby reinstating TREM2 expression and ameliorating sepsis-induced acute lung injury (SI-ALI) (60). In stark contrast, it has been documented that pro-inflammatory conditions, while suppressing TREM2 expression, concurrently diminish the expression levels of the STAT6 transcription factor downstream of the IL-4 pathway, thereby obstructing its anti-inflammatory capabilities (61). In a study focused on LPS-induced septic lung injury in mice, it was noted that the silencing of TREM2 could precipitate the downregulation of SHP1 and an increase in the phosphorylation of STAT3; conversely, the overexpression of TREM2 could mitigate oxidative stress and ferroptosis via the SHP1/STAT3 pathway, thus significantly alleviating sepsis-induced acute lung injury (62). Furthermore, the absence of TREM2 precipitates a dysregulation of macrophage function, exacerbating lung injury associated with sepsis, thereby indicating that the protective role of TREM2 in SI-ALI warrants considerable attention (63). Additionally, TREM2 deficiency has been shown to exacerbate kidney injury and LPS-induced acute lung injury in murine models (30). While TREM2 is widely acknowledged for its protective role in immune regulation, excessive activation of TREM2 may yield immunosuppressive effects, potentially impairing immune responses and heightening the risk of secondary infections following sepsis (64).

In summary, while TREM2 assumes a protective role in the modulation of immune responses during sepsis, its dysregulation may also culminate in adverse outcomes. This duality accentuates the significance of TREM2 in maintaining a delicate balance of pro-inflammatory signals, a crucial endeavor to avert excessive tissue damage in the context of sepsis. Thus, the intricate interplay of TREM2 in sepsis may herald a promising avenue for targeted therapeutic interventions aimed at the immune response in future endeavors.

## Strategies for TREM2 therapeutic agents in SI-ALI

As a pivotal element in immune regulation, the burgeoning interest in the development of pharmacological agents targeting TREM2 has become increasingly pronounced. Yet, the complexity inherent in the TREM2 signaling cascade, along with its unique mechanisms across various pathologies, renders the advancement and applicability of these therapies a formidable challenge. Currently, the majority of pharmacological agents directed at the TREM2 receptor are primarily examined within the context of Alzheimer's Disease (AD), with scant exploration in the realm of septic lung injury. Nevertheless, the insights derived from the investigation of TREM2-targeted therapies in AD may illuminate pathways for addressing septic lung injuries (Table 1).

**Table 1:** Research progress on TREM2-targeted drugs.

**Medicine**	**Mechanism**	**Disease**	**Development phase**	**References**
AL002	Anti-TREM2 McAb	Alzheimer	Clinical phase II (Terminated)	(66)
Iluzanebart (VGL101)	Anti-TREM2 McAb	Hereditary diffuse leukoencephalopathy combined with axonal spheroid degeneration; leukoencephalopathy	Clinical phase II	(73, 74)
VG-3927	TREM2 agonist	Alzheimer	Clinical phase I	(72)
VHB937	Anti-TREM2 McAb	Neurodegenerative disease; Amyotrophic lateral sclerosis	Preclinical	-
PY314	Anti-TREM2 McAb	Ovarian cancer; Gastric cancer; Colorectal cancer; Triple-negative breast cancer; Lung adenocarcinoma; Renal cell carcinoma	Clinical phase I	(70, 71)
DNL919	Anti-TREM2 McAb; TfR ligand	Alzheimer	Clinical phase I	(76)
Ab18 TVD-Ig/atfr	Anti-TREM2/TfR bispecific antibody	Alzheimer	Preclinical	(68)
ATV: TREM2	Transferrin receptor binding site antibody	Neurodegenerative disease	Preclinical	(69)
OPA	Small molecule TREM2 inhibitor	Colorectal cancer	Preclinical	(24)
68Ga-NOTA-COG1410	PET drugs labeled 68Ga; anti-TREM2 Polypeptide conjugated nuclides	PET imaging; Cancer	Preclinical	-

In Alzheimer's disease, the imperative to foster phagocytosis while mitigating inflammation emerges; however, the anti-inflammatory signaling of TREM2 may inadvertently disrupt the body's intrinsic anti-tumor defenses or exacerbate pro-fibrotic responses in the aftermath of liver injury (15). Thus, it becomes essential to strike a delicate balance in the activation of the TREM2 signaling pathway to attain the most favorable therapeutic outcomes. A selection of monoclonal agonistic anti-TREM2 antibodies has been crafted to amplify the protective transduction of TREM2 signals within microglia.

In the milieu of immune cells, the enzymes ADAM10 and ADAM17 engage in the cleavage of TREM2, resulting in the shedding of its extracellular domain and the release of soluble TREM2 (sTREM2), thereby culminating in the cessation of cell-autonomous signaling. The agonistic anti-TREM2 antibody, conceived from this understanding, is designed to bind to the upstream regions of the ADAM10 and ADAM17 cleavage sites, thwarting the release of sTREM2 and thereby promoting TREM2 signaling. In accordance with this mechanism, a poorly cleavable mutant of TREM2 has been engineered to enhance its presence on the cell surface, exhibiting efficacy akin to that of an agonistic antibody (65).

AL002, a humanized monoclonal antibody, has demonstrated the capacity to elevate cell survival and proliferation through the activation of TREM2 signaling, thereby counteracting dementia potentially arising from compromised TREM2 functionality, while simultaneously invigorating microglial activity. However, the prolonged administration of AL002 has been associated with an escalation in the dissemination of phosphorylated tau forms, which may, in turn, precipitate cognitive decline (66). Despite AL002 exhibiting a favorable pharmacokinetic/pharmacodynamic profile in healthy individuals, the drug has recently faltered in its ability to alleviate Alzheimer's symptoms during Alector's clinical trial.

Despite the promising therapeutic effects exhibited by currently available targeted drugs, it is noteworthy that only approximately 0.1% of antibodies for neurological disorders successfully traverse the blood-brain barrier and penetrate the brain parenchyma, necessitating the administration of significantly high antibody doses (67). Yet, recent investigations have unveiled an ingeniously engineered bispecific antibody, adeptly targeting both TREM2 and the transferrin receptor—designated as Ab18 TVD-Ig/aTfR—which remarkably amplifies the efficiency of central nervous system entry by over 10-fold, achieving a pervasive distribution throughout the brain parenchyma (68). These revelations serve as a poignant reminder that the tissue-specific delivery of pharmacological agents profoundly influences both therapeutic efficacy and safety. For instance, it is essential to deliberate whether the agents within SI-ALI can be judiciously directed toward the lungs to elicit pulmonary therapeutic effects, rather than being indiscriminately absorbed, thereby engendering unpredictable outcomes. Given that the majority of contemporary research remains ensconced within the confines of *in vitro* experimentation, uncertainty looms regarding the *in vivo* efficacy of these drugs and the ramifications of the body's complex physiological milieu on drug delivery.

Certain studies suggest that excessive stimulation of anti-TREM2 antibodies may perturb TREM2 expression within peripheral tissues, potentially undermining overall immune functionality. In response, researchers have devised an engineered antibody (ATV: TREM2) incorporating the transferrin receptor binding site to bolster endocytosis. Current investigations reveal that modest doses of this antibody yield improvements in amyloidosis. The distribution of ATV: TREM2 is contingent upon transferrin receptor expression, enabling its ingress into the spleen, bone marrow, plasma, brain, and other tissues to exert its influence. Moreover, this antibody is well-tolerated, exhibiting no deleterious effects on tissue integrity (69).

PY314, another humanized monoclonal antibody, functions as a TREM2 antagonist, effectively depleting tumor-associated macrophages. Clinical trial outcomes have indicated that PY314 is well-tolerated, suggesting that patients exhibit a favorable adaptation to treatment with this agent, accompanied by a reduction in adverse effects. This characteristic positions PY314 advantageously for broader applications within tumor therapy (70, 71). OPA, a platinum (IV)-based compound derived from Oxaliplatin (OP) and Artesunate (ART), has been shown to diminish TREM2 expression in macrophages, facilitating their polarization from an immunosuppressive M2 phenotype to an anti-tumor M1 phenotype. Furthermore, this compound fosters the maturation of dendritic cells, augments the functionality of natural killer (NK) cells, and amplifies the presence of CD8+ T cells within tumor tissues, thereby manifesting potent chemotherapeutic and immunostimulatory effects against cancer (24).

Furthermore, small molecule TREM2 activators such as VG-3927 (72) and humanized monoclonal antibody VGL101 (73, 74) are presently navigating the intricate landscape of clinical trials aimed at neurodegenerative disorders. Preliminary findings suggest that the synergistic combination of VGL101 with TREM2 engenders a selective activation of the receptor, thereby initiating a cascade of downstream signaling pathways that not only promote neuroprotection but also enhance the homeostatic functions of microglia. Notably, VG-3927 has demonstrated a significant reduction in soluble TREM2 levels within the cerebrospinal fluid of patients, illuminating its potential efficacy in clinical contexts targeting TREM2. Moreover, TREM2 antagonists may serve to alleviate neurotoxicity by tempering the excessive activation of microglia (75).

It is imperative to acknowledge that, despite the promising therapeutic effects exhibited by these agonists, no applicable drugs have yet attained approval. This underscores the necessity for further exploration, given the intricate mechanisms of action associated with the TREM2 target and its dual-sided effects, to thoroughly assess the safety and efficacy of its targeted pharmacological agents.

## Conclusion and prospect

TREM2 unveils a realm of promising potential in the treatment of sepsis-induced acute lung injury, its central role in immunomodulation and tissue repair establishing it as a formidable therapeutic target. Recent explorations have illuminated TREM2's dual contribution to the regulation of the immune system and its protective influence amidst the inflammatory tempest of sepsis. Thus, therapeutic strategies aimed at TREM2 may pave new pathways for the management of patients grappling with sepsis. Presently, the lion's share of mechanistic inquiries and drug development endeavors surrounding TREM2 predominantly fixates on neurological disorders. In stark contrast, our comprehension of its implications in sepsis-related maladies remains markedly less thorough. While this may appear as a daunting challenge, we can still draw upon the therapeutic effects and mechanisms of TREM2-targeted agents in other conditions to forge effective treatments for the intricate responses elicited by sepsis.

Although existing research has provided initial validation for TREM2's application in sepsis, it is imperative to acknowledge the potential variances in interpretations and outcomes across diverse studies. For instance, certain investigations may accentuate TREM2's capacity to mitigate the progression of lung injury, while others may underscore its multifaceted role in the nascent immune response. Therefore, future inquiries should endeavor to unravel the mechanisms governing TREM2's function, particularly its functional transformations across a spectrum of pathological states. Researchers ought to refine therapeutic approaches, honing in on the effective activation or inhibition of TREM2 signaling pathways to attain the most advantageous therapeutic outcomes.

While the prospect of targeted therapy involving TREM2 shines with promise, a profound investigation into the intricate signaling pathways is paramount to elucidate its mechanisms of action across diverse pathological states, as well as to evaluate the safety and efficacy of its clinical applications. Presently, a cadre of TREM2-specific pharmaceuticals is advancing through the stages of development and research. Notable among these, VG3927, VGL101, and VHB937 have exhibited encouraging therapeutic effects in clinical trials. Yet, the landscape remains devoid of any drug that has received approval and formal licensing for patient treatment. Consequently, ethical quandaries and barriers to clinical application loom as formidable challenges in TREM2-focused research. This prevailing scenario can be attributed to several pivotal factors. Firstly, investigations involving human genome editing and immunotherapy often incite ethical dilemmas, particularly in light of the uncertainties surrounding the long-term health ramifications for patients. Secondly, the design and execution of clinical trials must adhere to stringent ethical standards to ensure the comprehensive safeguarding of patients' informed consent and rights. Nevertheless, due to TREM2's intricate involvement in a multitude of diseases, researchers frequently find themselves navigating the delicate balance between scientific inquiry and ethical obligations. Furthermore, impediments to clinical application include the absence of effective biomarkers for monitoring TREM2 activity and its fluctuations during disease progression, resulting in a dearth of guidance for clinicians regarding the implementation of pertinent therapies.

It is well acknowledged that sepsis-related diseases encompass complex pathological processes, and the ensuing multi-organ damage presents significant challenges to treatment. TREM2, with its extensive array of ligands, exhibits functionality that is contingent upon the distribution of immune cells. Coupled with its diverse mechanisms of action, it undoubtedly injects uncertainty into the treatment of sepsis-related maladies. Thus, the pressing challenge remains: how to efficiently and specifically direct drugs to the intended organs while monitoring the site of drug action and effective concentration. The trajectory of TREM2-related research may be swayed by various patient-related factors. Therefore, it is imperative that forthcoming investigations not only address the scientific dimensions but also the ethical considerations to facilitate the clinical translation of TREM2.
